# Clinical and Research Implications of Nonspecific Necrosis on Peripheral Pulmonary Lesion Biopsies

**DOI:** 10.1016/j.chest.2025.09.015

**Published:** 2025-09-23

**Authors:** Alice Kennedy, Fabien Maldonado, Kaele Leonard, Taryn Boyle, Ankush Ratwani, Greta Bridwell, Jennifer D. Duke, Samira Shojaee, Rafael Paez, Robert J. Lentz

**Affiliations:** a Department of Internal Medicine, Vanderbilt University Medical Center, Nashville, TN; b Division of Allergy, Pulmonary, and Critical Care Medicine, Vanderbilt University Medical Center, Nashville, TN; c Department of Thoracic Surgery, Vanderbilt University Medical Center, Nashville, TN

To the Editor:

Necrosis is frequently identified in peripheral pulmonary lesion (PPL) biopsy specimens and is associated with various causes, notably malignancy and infection. A recent American Thoracic Society (ATS)/American College of Chest Physicians (CHEST) consensus definition of diagnostic yield, which is recommended for use in future diagnostic studies involving PPLs, classifies necrosis as a nonspecific (ie, nondiagnostic) finding.^1^ However, the significance of necrosis on biopsy and its diagnostic implications have not been well characterized.

## Methods

We analyzed a database of consecutive PPLs undergoing biopsy by either navigational bronchoscopy or transthoracic needle biopsy at Vanderbilt University Medical Center from 2017 to 2023. Histopathology and cytopathology reports were queried for the term *necrosis* and similar or overlapping terms (eg, *necrotic, necrotizing, caseating*). Specimens exhibiting necrosis were classified retrospectively using the ATS/CHEST diagnostic consensus definition as malignant, specific benign, or nonspecific benign based on pathologic findings. All PPLs were followed up for at least 1 year to determine the ultimate causes. The negative predictive value of nonspecific necrosis for an ultimately benign PPL was calculated, and nodule features between those determined to be benign vs malignant were compared using *t*-tests for continuous variables and χ^2^ tests for categorical variables. Analysis was conducted using R (version 4.4.1). This study was approved by the Vanderbilt University Medical Center institutional review board (IRB #212187).

## Results

**Figure 1:**
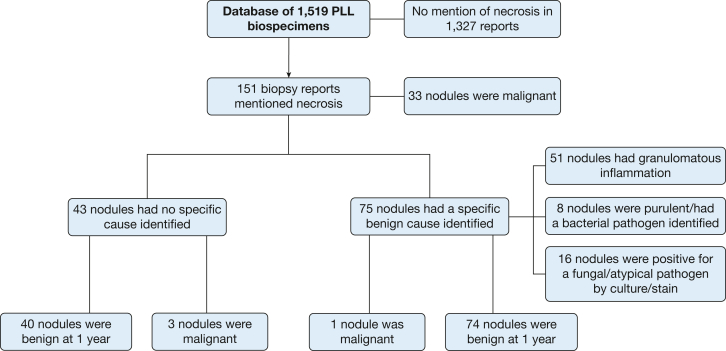
Flow diagram demonstrating process for inclusion and categorization. Specific terms used to query histopathology and cytopathology reports for inclusion in row 2 were necrosis, necrotic, necrotizing, and caseating. In row 3, specific as opposed to nonspecific necrosis was determined using the 2024 American Thoracic Society/American College of Chest Physicians consensus statement. The medical records for all included participants were reviewed after 1 year of follow-up to assess for the development of a malignancy after 1 year.

Among 1,519 biopsy specimens collected from 1,327 patients, 151 (10.0%) demonstrated necrosis. Specific diagnostic findings were present in addition to necrosis in 108 nodules, including malignancy in 33 (21.9%), granulomatous inflammation in 51 (33.8%), mycobacterial or fungal organism in 16 (10.6%), and bacterial culture growth in 8 (5.3%). A total of 43 (28.5%) cases exhibited necrosis without other specifically diagnostic findings (Fig 1). Three of 43 (7%) were ultimately determined to be malignant: 1 adenocarcinoma, 1 squamous cell carcinoma, and 1 metastatic renal cell carcinoma. Original pathology reports of both primary lung cancers described atypical cells suspicious but not diagnostic of malignancy in addition to necrosis. The negative predictive value of nonspecific necrosis for ultimately benign PPL origin was 93% (95% CI using the modified Wald method, 80.7%-98.3%). When only the single case of necrosis without atypia is considered, the negative predictive value estimate further increased to 97.6% (86.3%-100%).

Among PPLs with necrosis, those determined to be malignant were larger than those found to be benign (28.3 mm vs 21.6; mean difference, 6.6; 95% CI, 1.0-11.4; *P* < .03). See Table 1 for additional descriptive statistics regarding necrotic nodule radiographic features.

**Table 1:** Descriptive Statistics of Biopsied PPLs According to Pathologic Result

Characteristic	Benign necrosis, N = 114	Malignant necrosis, N = 37	No necrosis, N = 1368
Size in mm (SD)	21.60 (11.64)	28.25 (15.07)	19.59 (10.86)
Lung lobe			
LLL	16 (13.9%)	7 (19.4%)	229 (16.7%)
LUL	25 (21.7%)	15 (41.7%)	274 (20%)
Lingula	0 (0%)	2 (5.6%)	54 (3.9%)
RLL	19 (16.5%)	3 (8.3%)	281 (20.5%)
RML	14 (12.2%)	3 (8.3%)	118 (8.6%)
RUL	41 (35.7%)	6 (16.7%)	411 (30%)
Density			
Part-solid	7 (6.1%)	1 (2.8%)	204 (14.9%)
Pure ground-glass opacity	0 (0%)	0 (0%)	30 (2.2%)
Solid	108 (93.9%)	35 (97.2%)	1132 (82.7%)
Other radiographic features			
Spiculation	41 (35.7%)	12 (66.7%)	636 (46.5%)
Lobulated	28 (24.3%)	15 (41.7%)	318 (23.2%)
Smooth	11 (9.6%)	7 (19.4%)	120 (8.8%)
Cavitary	28 (24.3%)	5 (13.9%)	105 (7.7%)
Calcification	3 (2.6%)	2 (5.6%)	28 (2%)
Satellite micronodules	6 (5.2%)	0 (0%)	36 (2.6%)
TBNA/ FNA	108 (93.9%)	34 (94.4%)	1,243 (90.9%)
TBB/core	99 (86.1%)	30 (83.3%)	873 (63.8%)

Data are presented as No. (%) unless otherwise indicated. LLL = left lower lobe; LUL = left upper lobe; RLL = right lower lobe; RML = right middle lobe; RUL = right upper lobe; TBB/core = transbronchial biopsy/core; TBNA/ FNA = transbronchial needle aspiration/fine needle aspiration.

## Discussion

Necrosis was a common pathologic finding in this large cohort of PPL biopsies, noted in pathology reports of 10% of cases. Most cases of necrosis not associated with other specific pathologic findings were benign; among 43 PPLs with nonspecific necrosis on biopsy, only 3 (7%) were ultimately determined to be malignant. Notably, 2 of these 3 were accompanied by atypia suspicious for malignancy and were clinically managed as malignant nodules after index biopsy; a single case read as only nonspecific necrosis was ultimately determined to be malignant on re-biopsy after interval PPL growth on surveillance scan.

These results have important implications. From a clinical standpoint, PPLs yielding nonspecific necrosis without atypical cells are highly likely to be benign, particularly if small. However, because tumor necrosis is a well-described phenomenon, they should be monitored closely, and repeat biopsy may be considered if clinically indicated, especially in larger lesions with a continued high post-biopsy probability of malignancy.^2^^,^^3^

From a research standpoint, our results, if confirmed in larger studies, suggest that nonspecific necrosis in the absence of atypia may be considered diagnostic, contradicting the research consensus statement on diagnostic yield, which categorized necrosis as a nonspecific (nondiagnostic) finding.^1^ We have previously shown that this diagnostic yield construction is associated with very little discrepancy between yield (adjudicated as soon as pathology results return) and accuracy (informed by clinical follow-up allowing for an ultimate determination of nodule origin).^4^ When compared with the false-negative rate previously observed for specific benign findings on initial biopsy (1.9%), the rate of nonspecific necrosis without atypical cells later determined to be malignant (2.3%) in this study is similarly low. As such, there is a reasonable case to consider nonspecific necrosis absent atypia as a specific benign diagnosis, particularly if confirmed in additional studies.

This study has some limitations. It is a retrospective analysis with the usual limitations inherent in this study design. In particular, pathology was not reviewed prospectively with a focus on necrosis; it is possible that necrosis was not always noted in analyzed pathology reports, particularly when malignancy was also present. This could make our observed overall prevalence of necrosis (10%) an underestimate but would not affect findings related to nonspecific necrosis. Data were derived from a single center with a high rate of histoplasmosis, which may limit external validity; confirmation of these findings in larger, more geographically diverse cohorts will be important. Interrater differences in pathology interpretation were not evaluated. The single nonspecific necrosis case without atypia ultimately determined to be malignant did not undergo cytokeratin immunohistochemical staining, which might have detected atypia within this specimen had it been performed.

In conclusion, necrosis is a relatively common finding in PPL biopsy specimens. In those without additional malignant or specific benign findings, necrosis has a high negative predictive value for malignancy.

## Financial/Nonfinancial Disclosures

The authors have reported to *CHEST* the following: F. M. received compensation for services other than consulting from Intuitive Surgical and Medtronic; consulting payments from Intuitive Surgical, Johnson & Johnson, and Medtronic; education from Intuitive Surgical; and travel and lodging from Intuitive Surgical. He also received research support from Erbe Elektromedizin GmbH, Medtronic, and Steris Corporation. J. D. D. received consulting payments from Intuitive Surgical and travel and lodging from Intuitive Surgical. R. P. received consulting payments from Noah Medical. R. J. L. received education payments from Intuitive Surgical; consulting payments from Intuitive Surgical; and travel and lodging from Intuitive Surgical. None declared (A. K., K. M. L., T. B., A. R., G. B., and S. S.)
